# A new species of the genus *Pseudocrangonyx* (Crustacea, Amphipoda, Pseudocrangonyctidae) from Korea

**DOI:** 10.3897/zookeys.735.21697

**Published:** 2018-02-06

**Authors:** Chi-Woo Lee, Ko Tomikawa, Takafumi Nakano, Gi-Sik Min

**Affiliations:** 1 Department of Biological Sciences, Inha University, Incheon 22212, South Korea; 2 Department of Science Education, Graduate School of Education, Hiroshima University, Higashihiroshima 739-8524, Japan; 3 Present address: Department of Zoology, Graduate School of Science, Kyoto University, Kyoto 606-8502, Japan

**Keywords:** Crangonyctoidea, Korean Peninsula, interstitial water, molecular phylogeny

## Abstract

A new subterranean species of pseudocrangonyctid amphipod, *Pseudocrangonyx daejeonensis* **sp. n.** is described from the interstitial waters in Daejeon, Korea. *Pseudocrangonyx daejeonensis* **sp. n.** is distinguished from three morphologically similar congeners, *P. coreanus* Uéno, 1966, *P. febras* Sidorov, 2009, and *P. gudariensis* Tomikawa & Sato, 2016, by the characteristics of antenna 1, antenna 2, mandible, gnathopod 2, pleopods, uropods 1–2, and telson. Molecular phylogenetic analyses based on nuclear 28S rRNA and histone H3, and mitochondrial cytochrome *c* oxidase subunit I and 16S rRNA genes revealed that *P. daejeonensis* is a sister species of the unnamed *Pseudocrangonyx* sp. 3 inhabiting central Japan.

## Introduction

Amphipod species of the genus *Pseudocrangonyx* Akatsuka & Komai, 1922 have been known from subterranean waters and springs in Japan, the Korean Peninsula, Eastern China, and the Far East of Russia (Sidorov and Holsinger 2007; Tomikawa et al. 2016; Zhao and Hou 2017). Among the 22 known species of *Pseudocrangonyx*, only two species were recorded in Korean waters (Uéno 1966): *P. asiaticus* Uéno, 1934 and *P. coreanus* Uéno, 1966.

When Uéno (1966) described *P. coreanus* based on specimens collected from the Korean Peninsula, he clearly stated that morphological variations in the antennae, maxilla 1, uropod 3, and telson were observed among six populations of this species. Because recent systematic studies of *Pseudocrangonyx* in other regions have shown high species diversity within this genus (e.g., Tomikawa et al. 2016), it is highly possible that the true species diversity of *Pseudocrangonyx* amphipods inhabiting the Korean Peninsula remains under-estimated.

Recently, unidentified specimens of *Pseudocrangonyx* were collected during field surveys of interstitial invertebrates in Korea by the first author. In this paper, we describe and illustrate this amphipod as a new species. In addition, the phylogenetic position of the new species was estimated using nuclear 28S rRNA and histone H3, and mitochondrial cytochrome *c* oxidase subunit I (COI) and 16S rRNA sequence data.

## Materials and methods

### Sampling

**Figure 1.**
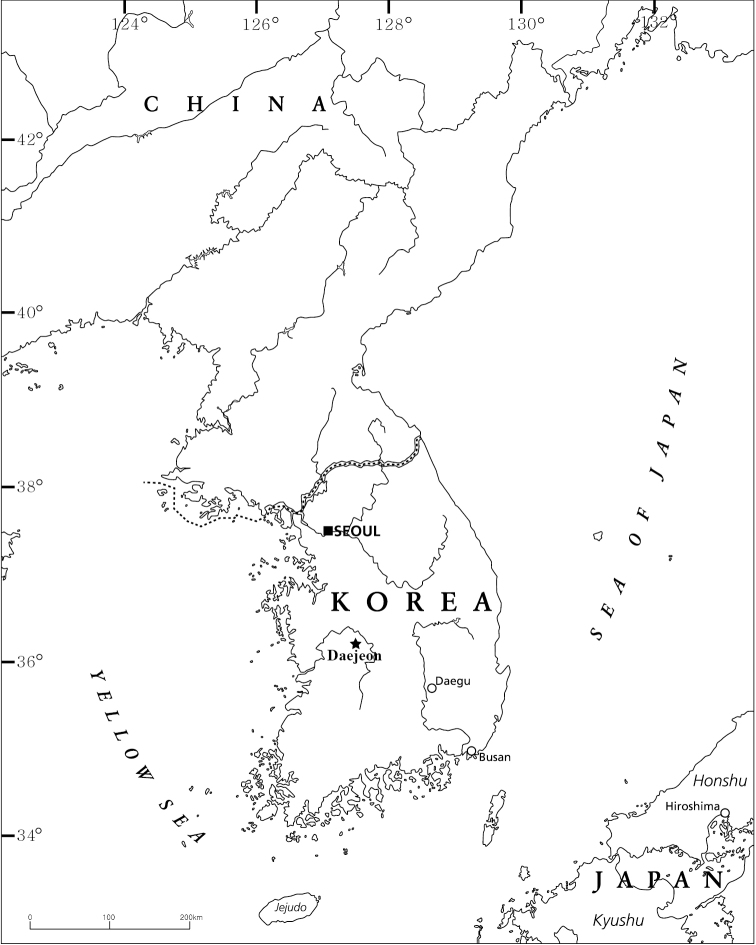
Map showing the collection locality of the specimens examined in this study.

*Pseudocrangonyx* specimens were collected from interstitial water in Heukseok-dong, Seo-gu, Daejeon, South Korea (Fig. 1) using a 50 μm fine-mesh net. Specimens were pumped up with 80–100 L of interstitial water at 1–1.5 m beneath hyporheic zones using a core (Lee and Park 2016). All specimens were immediately preserved in 95% ethanol. The specimens are deposited in the collection of the Nakdonggang National Institute of Biological Resources, Korea (NNIBR) and in the Zoological Collection of Kyoto University (KUZ).

### Morphological observation

The specimens were dissected in 70 % ethanol and mounted in gum-chloral medium on glass slides under a stereomicroscope (Model SZX-7; Olympus, Tokyo, Japan). Specimens were examined using a Nikon Eclipse Ni light microscope (Nikon, Tokyo, Japan) and illustrated with the aid of a drawing tube. The body length from the tip of the rostrum to the base of the telson was measured along the dorsal curvature to the nearest 0.1 mm. The nomenclature of the setal patterns on the mandibular palp follows that of Stock (1974).

### Molecular phylogenetic analyses

Methods of the genomic DNA extraction, PCR and DNA sequencing were performed following Tomikawa et al. (2016). Accordingly, nine DNA sequences of nuclear 28S rRNA, histone H3, COI and 16S rRNA from three Korean *Pseudocrangonyx* specimens were newly obtained in this study, and deposited into the International Nucleotide Sequence Database Collaboration (INSDC) through the DNA Data Bank of Japan (Table 1).

**Table 1.** Samples used for the phylogenetic analyses. The information on the vouchers is accompanied by the collection locality and the INSDC accession numbers. Sequences marked with an asterisk were obtained for the first time in the present study. Acronyms: IZCASIZCAS, Institute of Zoology, Chinese Academy of Sciences; NNIBR, Nakdonggang National Institute of Biological Resources; NSMT, National Museum of Nature and Science, Tokyo.

Species	Voucher or isolate #	Locality or country	INSDC #			
			28S	Histone H3	COI	16S
*Pseudocrangonyx*						
*P. daejeonensis* sp. n.	NNIBRIV1 (Holotype)	Daejeon, Korea	LC322136*	LC322138*	LC322137*	LC322135*
*P. daejeonensis* sp. n.	NNIBRIV2 (Paratype)	Daejeon, Korea		LC322143*		
*P. daejeonensis* sp. n.	NNIBRIV3 (Paratype)	Daejeon, Korea	LC322140*	LC322142*	LC322141*	LC322139*
*P. gudariensis*	NSMT-Cr 24605	Aomori, Japan	LC171498	LC171500	LC171499	LC171497
*P. yezonis*	G1280	Hokkaido, Japan	LC171518	LC171520	LC171519	LC171517
*P. yezonis*	G1279	Akita, Japan	LC171514	LC171516	LC171515	LC171513
*Pseudocrangonyx* sp. 1	G400	Iwate, Japan				LC171479
*Pseudocrangonyx* sp. 1	G1281	Iwate, Japan				LC171521
*Pseudocrangonyx* sp. 2	G1283	Okayama, Japan	LC171525	LC171527	LC171526	LC171524
*Pseudocrangonyx* sp. 2	G1277	Yamaguchi, Japan	LC171506	LC171508	LC171507	LC171505
*Pseudocrangonyx* sp. 2	G1278	Yamaguchi, Japan	LC171510	LC171512	LC171511	LC171509
*Pseudocrangonyx* sp. 3	G404	Shiga, Japan	LC171488	LC171489		
*Pseudocrangonyx* sp. 3	G405	Shiga, Japan	LC171491	LC171493	LC171492	LC171490
*Pseudocrangonyx* sp. 3	G406	Shiga, Japan	LC171495	LC171496		LC171494
*Pseudocrangonyx* sp. 4	G1282	Shiga, Japan		LC171523		LC171522
*Pseudocrangonyx* sp. 5	G402	Shimane, Japan	LC171485	LC171487	LC171486	LC171484
*Pseudocrangonyx* sp. 5	G401	Shimane, Japan	LC171481	LC171483	LC171482	LC171480
*Pseudocrangonyx* sp. 5	G1271	Kagawa, Japan	LC171502	LC171504	LC171503	LC171501
*Pseudocrangonyx* sp. 5	G1295	Kochi, Japan	LC171533	LC171535	LC171534	LC171532
*Pseudocrangonyx* sp. 5	G1296	Kochi, Japan	LC171537	LC171539	LC171538	LC171536
*Pseudocrangonyx* sp. 5	G1294	Ehime, Japan	LC171529	LC171531	LC171530	LC171528
*Pseudocrangonyx* sp. 6	G1297	Gifu, Japan	LC171541	LC171543	LC171542	LC171540
*P. holsingeri*		Russian Far East	KJ871679		KF153111	
*P. korkishkoorum*	B1	Russian Far East	KJ871678		KF153107	
*P. korkishkoorum*	B2	Russian Far East			KF153108	
*P. korkishkoorum*	B3	Russian Far East			KF153109	
*P. korkishkoorum*	N1	Russian Far East	KJ871676		KF153105	
*P. korkishkoorum*	N2	Russian Far East	KJ871677		KF153106	
*P. kseniae*		Russian Far East	KJ871675		KF153115	
*P. susanaensis*		Russian Far East			KF153113	
*P. sympatricus*		Russian Far East			KF153112	
*P. tiunovi*		Russian Far East	KJ871674		KF153110	
*P. elegantulus*	IZCASIZCAS I-A1602-2	China	KY436646		KY436647	
Outgroup						
*Crymostygius thingvallensis*						HQ286009
*Eocrangonyx primoryensis*						HQ286011
*Crangonyx floridanus*	G1322	Chiba, Japan	LC171549		LC171550	LC171548

The OTU set for phylogenetic analyses was almost identical to that used in the previous phylogenetic analyses in Tomikawa et al. (2016) with the DNA sequences of *P. elegantulus* Hou in Zhao and Hou (2017) (Table 1). The alignments of H3 and COI were trivial, as no indels were observed. 28S and 16S sequences were aligned using MAFFT v. 7.310 (Katoh and Standley 2013). The lengths of the 28S, H3, COI and 16S were 1357, 328, 658, and 430 bp, respectively. The concatenated sequences thus yielded 2773 bp of alignment positions. Phylogenetic trees were constructed using maximum likelihood (ML) and Bayesian inference (BI). The ML phylogeny was constructed using RAxML v. 8.2.8 (Stamatakis 2014) with the substitution model set as GTRCAT, immediately after nonparametric bootstrapping (BS) conducted with 1000 replicates. The best-fit partition scheme was identified with Akaike information criterion using PartitionFinder v. 2.1.1 (Lanfear et al. 2017) with the “greedy” algorithm (Lanfear et al. 2012): 28S/1st and 2nd positions of H3/H3 3rd position/COI 1st position/COI 2nd position/COI 3rd position/16S. BI and Bayesian posterior probabilities (PPs) were estimated using MrBayes v. 3.2.6 (Ronquist et al. 2012). The best-fit partition scheme and models for each partition were selected based on the Bayesian information criterion using PartitionFinder with the “greedy” algorithm: for 28S, GTR+G; for H3 1st and 2nd position, JC+I; for H3 3rd position, K80+G; for COI 1st position, SYM+I+G; for COI 2nd position, F81+I; for COI 3rd position, GTR+I+G; and GTR+I+G for 16S. Two independent runs of four Markov chains were conducted for 20 million generations, and the tree was sampled every 100 generations. The parameter estimates and convergence were checked using Tracer v. 1.6.0 (Rambaut and Drummond 2013), and the first 50001 trees were discarded based on the results.

## Taxonomy

### Family Pseudocrangonyctidae Holsinger, 1989

#### Genus *Pseudocrangonyx* Akatsuka & Komai, 1922

##### Pseudocrangonyx daejeonensis sp. n.

Taxon classificationAnimaliaORDOFAMILIA

http://zoobank.org/ECC7F708-DD43-4A48-9458-B6DA59265796

Figs 2 , 3 , 4 , 5 , 6 , 7 , 8 , 9

###### Material examined.

Holotype: Female (NNIBRIV1, 3.8 mm), Heukseok-dong (36°15.65'N, 127°20.43'E), Daejeon, Korea, collected by Lee CW on 31 May 2017. Paratypes: 1 male (NNIBRIV2, 2.7 mm), 1 female (NNIBRIV3, 2.3 mm), 3 females (KUZ Z1924), data same as for holotype.

**Etymology.** The specific name is an adjective derived from the type locality name of the new species.

###### Description.

**Figure 2.**
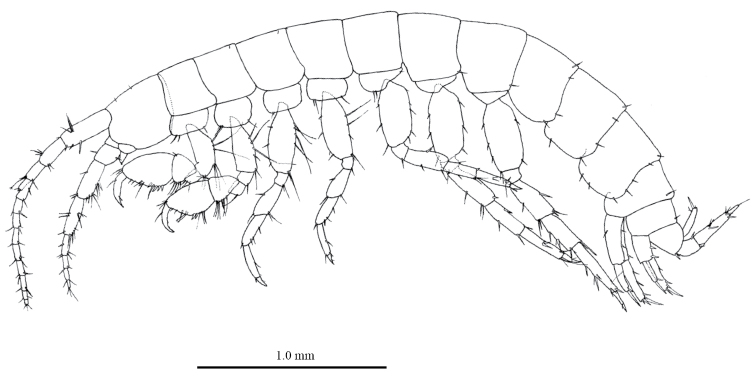
*Pseudocrangonyx daejeonensis* sp. n., holotype, female (3.8 mm), NNIBRIV1. Habitus, lateral view.

*Female* [NNIBRIV1, 3.8mm]. Head (Fig. 2) with short dorsal setae; rostrum reduced; lateral cephalic lobe rounded; antennal sinus shallow with rounded angle; eyes absent. Dorsal margin of pleonites 1–3 and urosomites 1–2 with setae (Fig. 2). Ventral margin of urosomite 1 without setae (Fig. 2). Ventral margin of epimeral plate 1 with seta, posteroventral corner rounded with seta (Fig. 2); ventral and posterior margins of plate 2 each with seta, posteroventral corner rounded with seta (Fig. 2); ventral and posterior margins of plate 3 each with 2 setae, posteroventral corner rounded with seta (Fig. 2).

**Figure 3.**
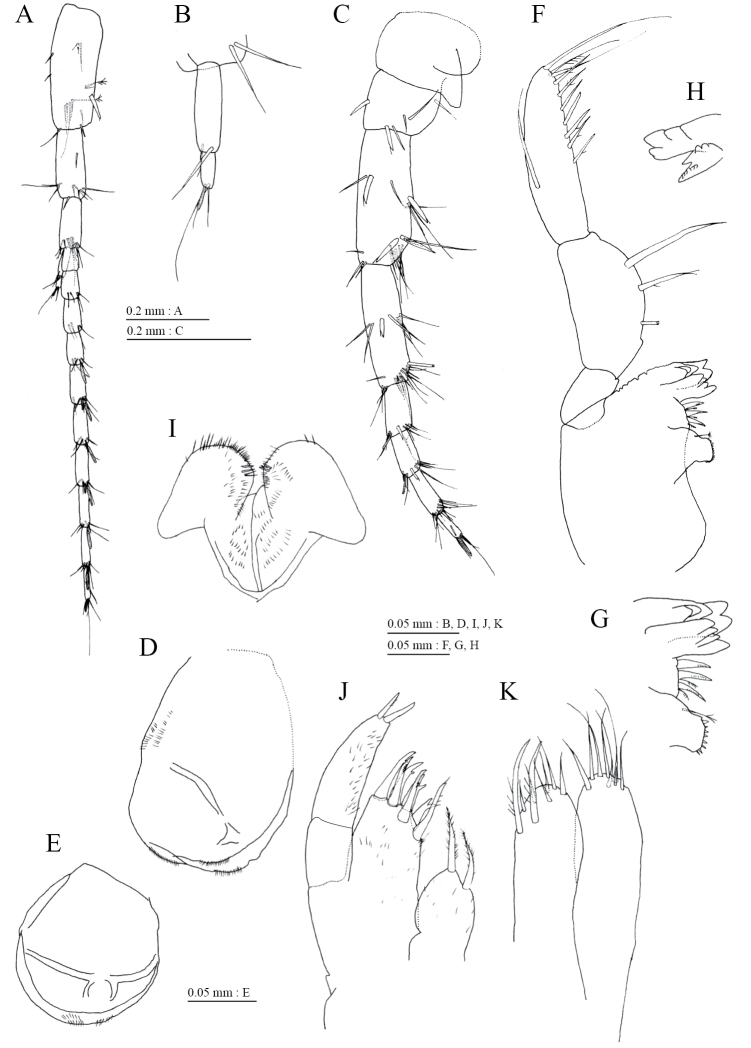
*Pseudocrangonyx daejeonensis* sp. n., holotype, female (3.8 mm): **A–D, F–K** paratype female (2.3 mm): **E**. **A** antenna 1, lateral view **B** accessory flagellum of antenna 1, lateral view **C** antenna 2, medial view **D** upper lip, anterior view **E** upper lip, anterior view **F** left mandible, medial view **G** incisor, lacinia mobilis, and molar process of left mandible, medial view **H** incisor and lacinia mobilis of right mandible, medial view **I** lower lip, ventral view **J** maxilla 1, dorsal view **K** maxilla 2, ventral view.

Antenna 1 (Fig. 3A) 0.38 times as long as body length, peduncular articles 1 to 3 in length ratio of 1.0 : 0.5 : 0.4; accessory flagellum (Fig. 3B) 2-articulate, terminal article with 3 setae and 1 aesthetasc; primary flagellum 10-articulate, 1 aesthetasc on some articles. Antenna 2 (Fig. 3C) 0.58 times as long as antenna 1; flagellum 0.65 times as long as peduncular articles 4 and 5 combined, consisting of 4 articles; calceoli absent.

**Figure 4.**
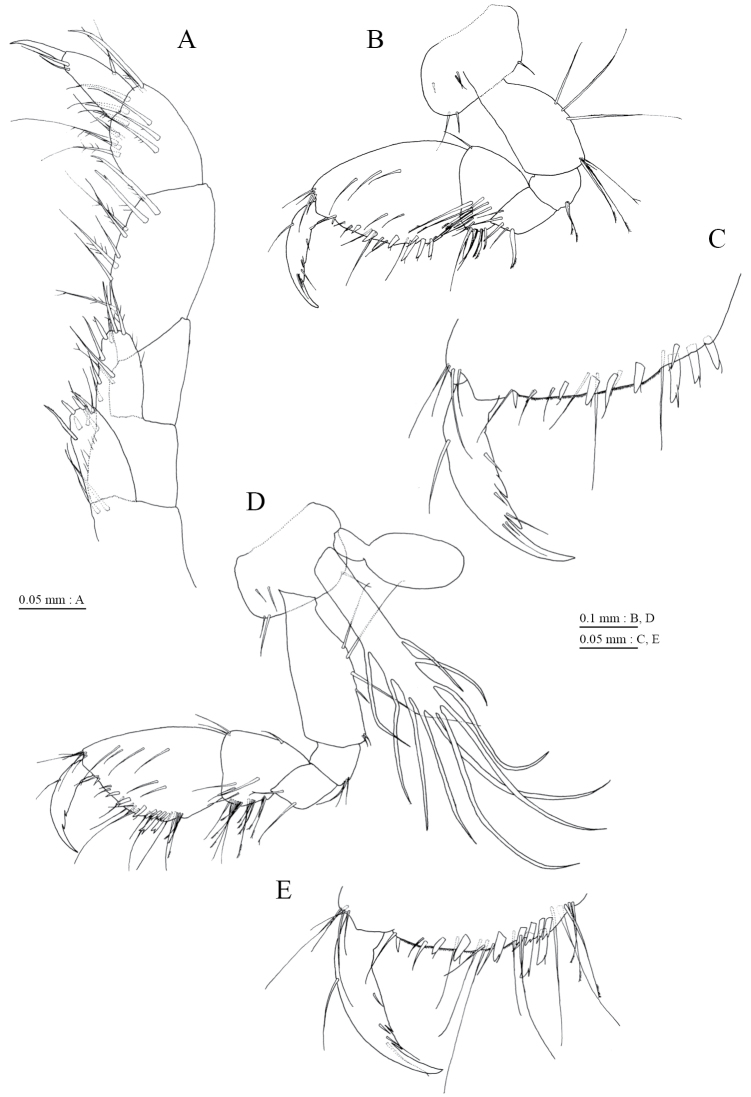
*Pseudocrangonyx daejeonensis* sp. n., holotype, female (3.8 mm). **A** maxilliped, dorsal view **B** gnathopod 1, medial view **C** palmar margin of propodus and dactylus of gnathopod 1, medial view **D** gnathopod 2, medial view **E** palmar margin of propodus and dactylus of gnathopod 2, medial view.

Upper lip (Fig. 3D) with rounded anterior margin, bearing fine setae. Mandibles (Fig. 3F, G, H) with left and right incisors with 5- and 4-dentate, respectively; left lacinia mobilis 4-dentate, right lacinia bifid, bearing many teeth; molar process triturative; accessory setal rows of left and right mandibles with 3- and 2- pectinate setae, respectively; palp 3-articulate, article 3 with 1 A-, 7 D-, and 3 E-setae. Lower lip (Fig. 3I) with broad outer lobes with fine setae, mandibular process of outer lobe rounded apically; inner lobes indistinct. Maxilla 1 (Fig. 3J) with inner and outer plates, and palp; inner plate subovate with 2 plumose setae; outer plate subrectangular with 7 serrate teeth apically; palp 2-articulate, longer than outer plate, article 2 with 2 apical robust setae. Maxilla 2 (Fig. 3K) with oblique inner row of 2 setae on inner plate. Maxilliped (Fig. 4A) with inner and outer plates, and palp; inner plate reaching base of palp article 1, with 2 apical robust setae; outer plate not exceeding end of palp article 1, with 2 plumose setae and some medial setae; palp 4-articulate, medial margin of article 2 lined with setae, article 4 with nail.

**Figure 5.**
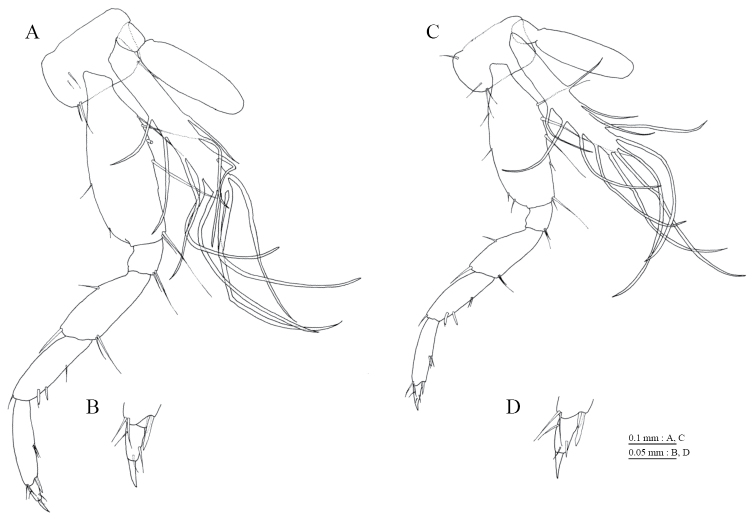
*Pseudocrangonyx daejeonensis* sp. n., holotype, female (3.8 mm). **A** pereopod 3, medial view **B** dactylus of pereopod 3, medial view **C** pereopod 4, medial view **D** dactylus of pereopod 4, medial view.

**Figure 6.**
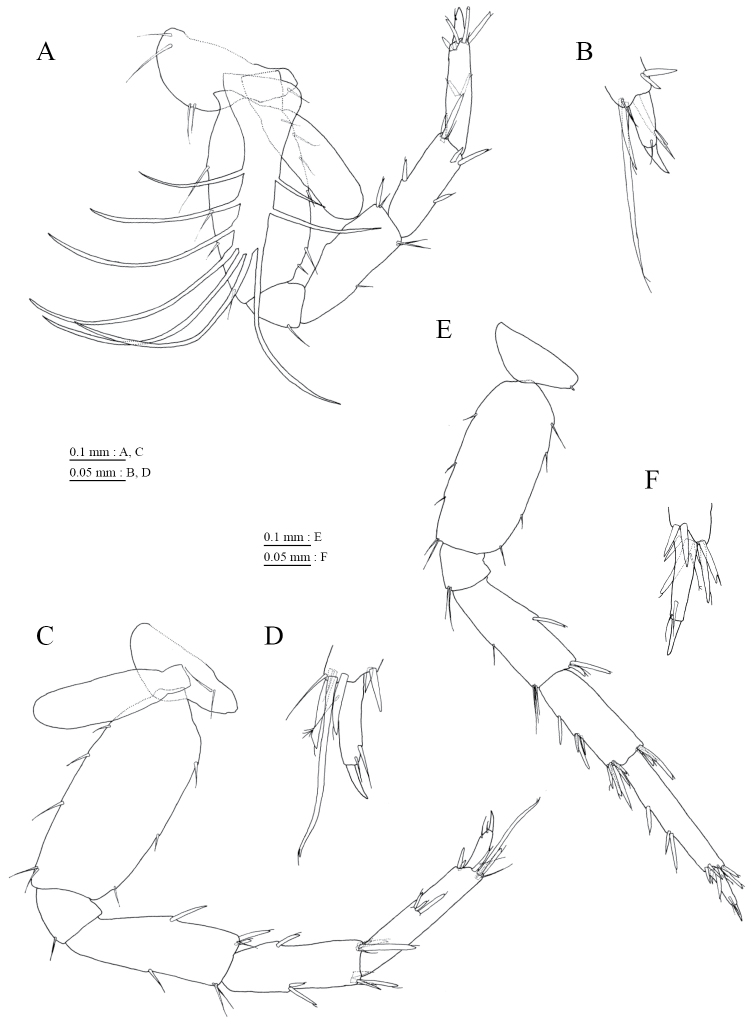
*Pseudocrangonyx daejeonensis* sp. n., holotype, female (3.8 mm). **A** pereopod 5, medial view **B** dactylus of pereopod 5, medial view **C** pereopod 6, medial view **D** dactylus of pereopod 6, medial view **E** pereopod 7, lateral view **F** dactylus of pereopod 7, lateral view.

Gnathopod 1 (Fig. 4B) with subquadrate coxa, bearing setae on its anterodistal and posteroventral corners, width 1.9 times as long as depth; posterior margin of basis with 3 setae; posterodistal corner of carpus with slender setae, some weakly pectinate; propodus stout, subchelate, palmar margin with 3 medial and 3 lateral robust setae; posterior margin of dactylus dentate (Fig. 4C). Gnathopod 2 (Fig. 4D) with subquadrate coxa, bearing setae on its anterodistal and posteroventral corners, width 1.6 times as long as depth; posterior margin of basis with 4 setae; posterodistal corner of carpus with slender setae, some weakly pectinate; propodus stout, subchelate, palmar margin with 7 medial and 2 lateral robust setae; posterior margin of dactylus dentate (Fig. 4E). Pereopod 3 (Fig. 5A) with subquadrate coxa bearing setae on its anterodistal and posteroventral corners, width 1.6 times as long as depth; anterior and posterior margins of basis with 2 and 4 setae, respectively; merus, carpus, and propodus in length ratio of 1.0 : 0.9 : 0.8; posterior margin and submargin of dactylus each with seta (Fig. 5B). Pereopod 4 (Fig. 5C) with subquadrate coxa bearing setae on its anterodistal corner, width 1.7 times as long as depth; anterior and posterior margins of basis each with 4 setae; merus, carpus, and propodus in length ratio of 1.0 : 0.7 : 0.8; posterior margin and submargin of dactylus each with seta (Fig. 5D). Pereopod 5 (Fig. 6A) with weakly bilobed coxa bearing setae on anterior and posterior lobes, width 1.7 times as long as depth; anterior and posterior margins of basis with 4 and 6 setae, respectively; merus, carpus, and propodus in length ratio of 1.0 : 0.7 : 0.8; anterior margin of dactylus with 2 setae (Fig. 6B). Pereopod 6 (Fig. 6C) with coxa bearing concave lower margin, marginally bare; anterior and posterior margins of basis with 5 and 3 setae, respectively; merus, carpus, and propodus in length ratio of 1.0 : 0.8 : 0.9; anterior margin of dactylus with 2 setae (Fig. 6D). Pereopod 7 (Fig. 6E) with subtriangular coxa, bearing seta on posteroproximal corner; anterior and posterior margins of basis with 3 and 4 setae, respectively; merus, carpus, and propodus in length ratio of 1.0 : 0.8 : 1.1; anterior margin of dactylus with 2 setae (Fig. 6F).

Coxal gills (Figs 4D, 5A, C, 6A, C) on gnathopod 2 and pereopods 3–6; sternal gills absent. Brood plates (Figs 4D, 5A, C, 6A) slender, with numerous setae, on gnathopod 2 and pereopods 3–5.

**Figure 7.**
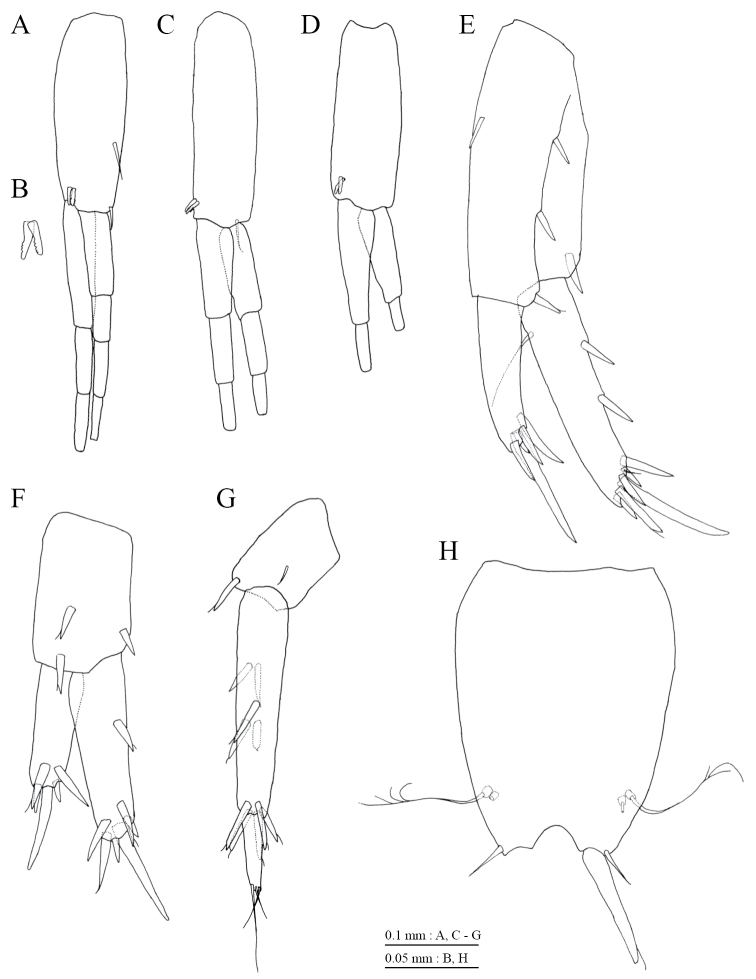
*Pseudocrangonyx daejeonensis* sp. n., holotype, female (3.8 mm). **A** pleopod 1, anterior view **B** retinacula on peduncle of pleopod 1, anterior view **C** pleopod 2, anterior view **D** pleopod 3, anterior view **E** uropod 1, dorsal view **F** uropod 2, ventral view **G** uropod 3, dorsal view **H** telson, ventral view. Plumose setae on pleopodous rami omitted.

Peduncle of pleopod 1 (Fig. 7A) with 1 outer marginal and 1 outerdistal seta; peduncle of pleopod 2 (Fig. 7C) with outerdistal seta; peduncle of pleopod 3 (Fig. 7D) lacking marginal and distal setae. Pleopods 1–3 with paired retinacula (Fig. 7B), and lacking bifid setae (clothes-pin setae) on inner basal margin of inner ramus; inner ramus of pleopods 1–3 3-, 3-, and 2-articulate (Fig. 7A, C, D); outer ramus of pleopods 1–3 4-, 3-, and 2-articulate (Fig. 7A, C, D).

Uropod 1 (Fig. 7E) with basofacial seta on peduncle; inner ramus 0.87 times as long as peduncle, inner margin of former with 2 robust setae, outer margin bare, basal part with slender seta; outer ramus 0.63 times as long as inner, marginally bare. Uropod 2 (Fig. 7F) with inner and outer rami; inner ramus 1.10 times as long as peduncle, its inner margin with robust seta, outer margin without setae; outer ramus 0.68 times as long as inner ramus, marginally bare. Uropod 3 (Fig. 7G) with peduncle 0.34 times as long as outer ramus, with 1 robust and 1 slender setae; inner ramus absent; outer ramus 2-articulate, proximal article with robust setae, terminal article 0.32 times as long as proximal article, with 3 distal setae. Telson (Fig. 7H) length 1.3 times as long as wide, cleft for 0.08 times of length, each telson lobe with 2 lateral penicillate setae, 1 apical robust and 1 apical short setae.

**Figure 8.**
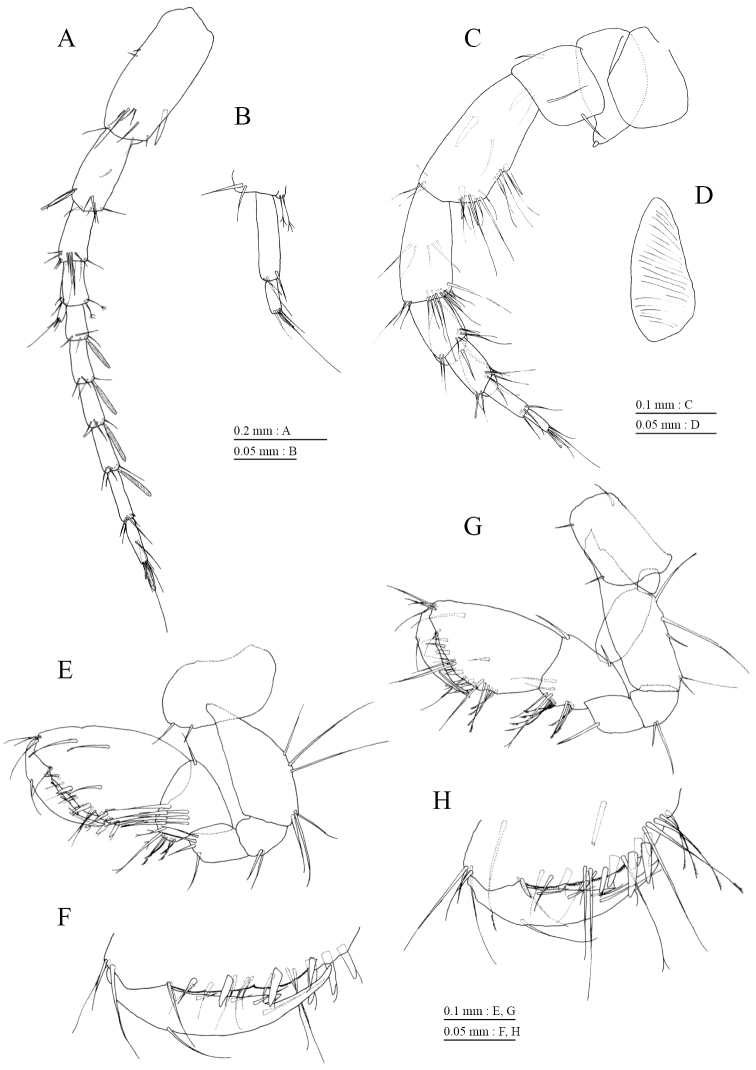
*Pseudocrangonyx daejeonensis* sp. n., paratype, male (2.7 mm). **A** antenna 1, lateral view **B** accessory flagellum of antenna 1, medial view **C** antenna 2, lateral view **D** calceolus of antenna 2, medial view **E** gnathopod 1, medial view **F** palmar margin of propodus and dactylus of gnathopod 1, medial view **G** gnathopod 2, lateral view **H** palmar margin of propodus and dactylus of gnathopod 2, lateral view.

*Male* [NNIBRIV2, 2.7 mm]. Antenna 1 (Fig. 8A, B) 0.46 times as long as body length, primary flagellum 7-articulate, 1 aesthetasc on some articles. Antenna 2 (Fig. 8C) 0.57 times as long as antenna 1; flagellum 0.72 times as long as peduncular articles 4 and 5 combined, consisting of 4 articles, first 2 of which with calceoli (Fig. 8D).

Gnathopod 1 (Fig. 8E) with coxa width 1.84 times as long as depth; palmar margin with 3 medial and 3 lateral robust setae (Fig. 8F). Gnathopod 2 (Fig. 8G) with coxa width 1.66 times as long as depth; palmar margin with 3 medial and 4 lateral robust setae (Fig. 8H).

**Figure 9.**
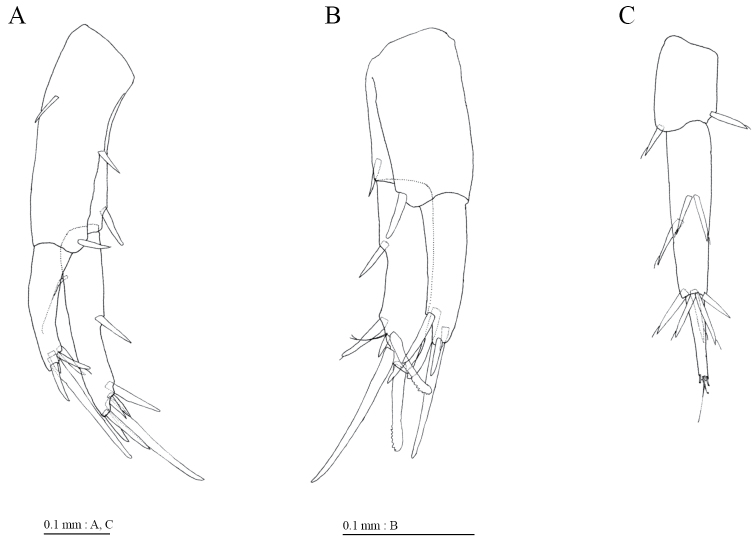
*Pseudocrangonyx daejeonensis* sp. n., paratype, male (2.7 mm). **A–C** uropods 1–3, respectively, dorsal views.

Uropod 1 (Fig. 9A) with robust seta on inner margin of inner ramus; outer ramus 0.62 times as long as inner. Uropod 2 (Fig. 9B) with 2 serrate and 4 simple robust setae and slender seta at distal part. Uropod 3 (Fig. 9C) with peduncle 0.32 times as long as outer ramus; terminal article of outer ramus 0.5 times as long as proximal article.

**Variation.** Peduncle of pleopod 1 with or without seta on outer margin.

###### Distribution.

This species is known only from the type locality.

###### Molecular phylogenetic position.

**Figure 10.**
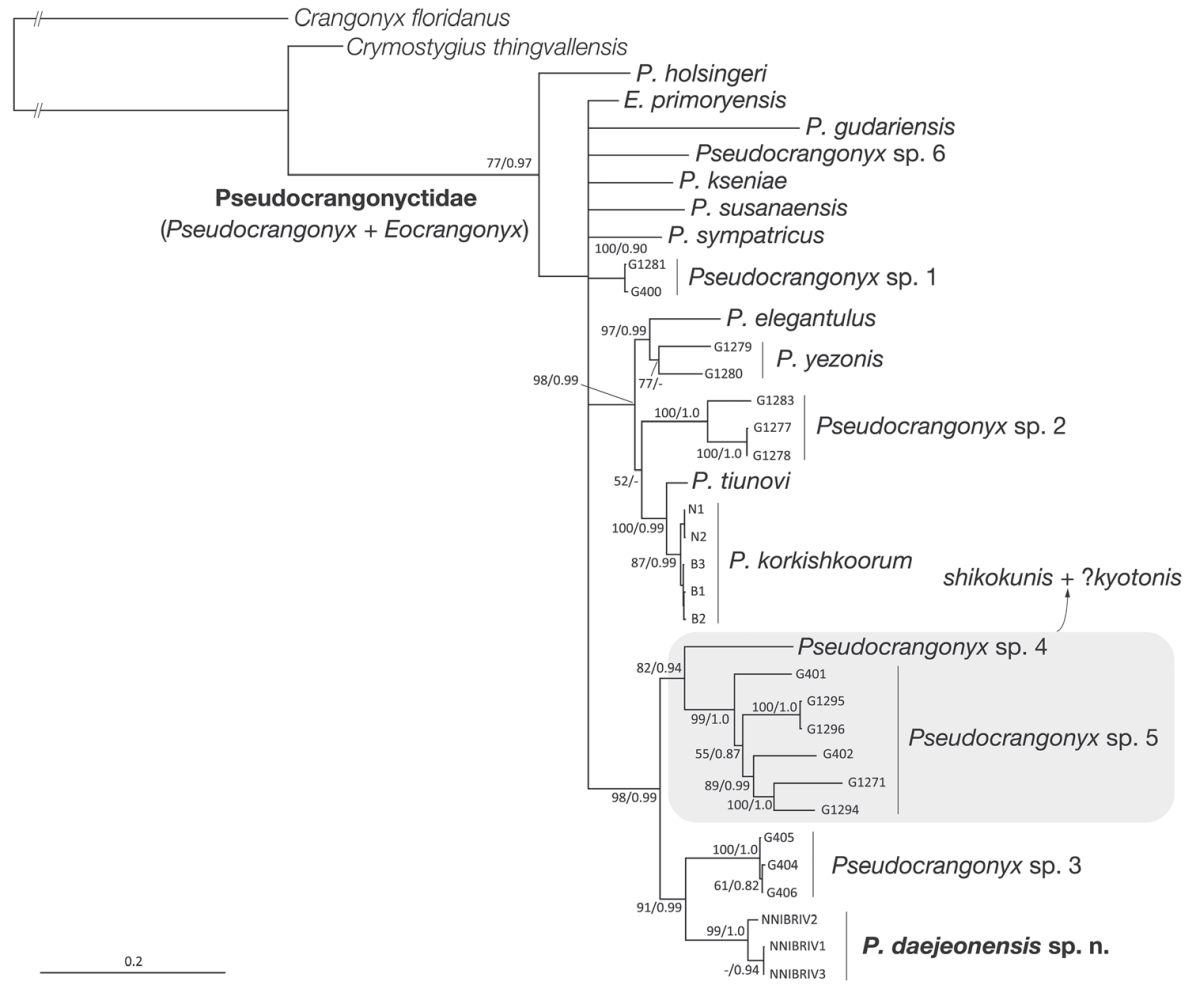
Bayesian inference tree for 2773 bp of nuclear 28S rRNA, plus histone H3 and mitochondrial COI and 16S rRNA markers. Numbers on nodes represent bootstrap values for maximum likelihood and Bayesian posterior probabilities. Specimen numbers are shown in Table 1.

The BI tree (mean ln *L* = −14039.10; Fig. 10) for estimating the phylogenetic position of the new species had an identical topology to that of the ML tree (ln *L* = −14504.12; not shown). *Pseudocrangonyx daejeonensis* belonged to a well-supported clade (BS = 98 %, PP = 0.99) containing the three phylogroups known from the western parts of Honshu and Shikoku, i.e., *Pseudocrangonyx* spp. 3–5. The new species formed a clade (BS = 91 %, PP = 0.99) with *Pseudocrangonyx* sp. 3 inhabiting the eastern part of Shiga Prefecture, Japan. Monophyly of the present specimens of *P. daejeonensis* was fully-supported (BS = 99 %, PP = 1.0).

###### Remarks.

*Pseudocrangonyx daejeonensis* is morphologically similar to *P. coreanus* in having 1) relatively small body size (smaller than 6 mm), 2) eyes completely absent, 3) carpus of gnathopod 2 without serrate robust setae on posterodistal corner, 4) outer margin or outer distal corner of pleopods 1 and 2 with setae, 5) inner basal margin of inner ramus of pleopods without bifid setae, and 6) small number of articles (less than 5) of rami of pleopods. However, the former is distinguished from the latter by the following features (features of *P. coreanus* in parentheses): 1) antenna 1 shorter (longer) than 0.4 times as long as body length, 2) antenna 2 of female without calceoli (with calceoli), 3) uropod 1 not exceeding (slightly exceeding) tip of uropod 2, and 4) outer ramus of uropod 2 without robust seta (with robust seta).

*Pseudocrangonyx daejeonensis* is also similar to *P. febras* Sidorov, 2009 and *P. gudariensis* Tomikawa and Sato in Tomikawa et al. (2016) in having 1) relatively smaller body size, 2) eyes completely absent, and 3) urosomite 1 without basal setae. However, *P. daejeonensis* is distinguished from these two species by the following features: from *P. febras* (features of *P. febras* in parentheses), 1) antenna 1 shorter than 0.4 times as long as body length (longer than 0.7 times), 2) peduncular article 2 of antenna 1 0.5 (0.7) times as long as article 1, 3) palp article 2 of mandible with 3 (7) setae, 4) carpus of male gnathopod 2 without serrate robust setae on posterodistal corner (with serrate robust setae), 5) fewer articles of pleopodal rami, up to 4 (more, up to 6), 6) inner ramus of uropod 1 with 2 inner marginal robust setae (5 inner and 3 outer marginal robust setae), 7) outer ramus of uropod 1 without setae (with 2 robust setae), and 8) inner ramus of uropod 2 with inner robust seta (3–4 inner and 2–3 outer marginal robust setae); from *P. gudariensis* (features of *P. gudariensis* in parentheses), 1) basal part of inner ramus of uropod 1 with 1 slender setae (with 3 slender setae), 2) outer ramus of uropod 1 without setae (with 2 setae), 3) inner margin of inner ramus uropod 2 with 1 robust setae (with 4 robust setae), and 4) telson lobe with 1 robust seta apically (with 2 robust setae apically).

Although the phylogenetic position of *P. coreanus* remains uncertain, the results of the previous molecular phylogenetic studies (Tomikawa et al. 2016; Zhao and Hou 2017) and our phylogenetic analyses showed that *P. daejeonensis* and the two morphologically similar species, *P. febras* and *P. gudariensis*, did not form a monophyletic lineage with large genetic divergences. Because these three species inhabit interstitial waters, not subterranean habitats, morphological similarities observed among them may reflect their similar habitat preferences.

The phylogenetic position of *P. daejeonensis* also sheds light onto the complex faunistic relationships between the *Pseudocrangonyx* species inhabiting the Japanese Archipelago and those inhabiting the Far Eastern continental area. Common ancestors of the Japanese *Pseudocrangonyx* species were considered to have migrated from the continental part to the Japanese Archipelago (Sidorov and Holsinger 2007). Previous systematic studies revealed that the *Pseudocrangonyx* amphipods distributed in northern Japan and the western tip of Honshu, Japan, i.e., *P. yezonis* and *Pseudocrangonyx* sp. 2, are phylogenetically close to the continental species (Sidorov and Holsinger 2007; Tomikawa et al. 2016; Zhao and Hou 2017). As *P. daejeonensis* formed a well-supported clade with *Pseudocrangonyx* sp. 3, which is indigenous to the central part of Honshu, their phylogenetic relationship suggested that the species diversity of the Japanese *Pseudocrangonyx* has been increased as a result of multiple continental-origins. It is also feasible that *P. daejeonensis* diverged from a common ancestor indigenous to the Japanese Archipelago. To clarify the biogeographical history of *Pseudocrangonyx* amphipods, further faunistic surveys along with molecular phylogenetic analyses are essentially needed.

The uncorrected *p*-distance of 15.0 % for COI, calculated using MEGA7.0.16 (Kumar et al. 2016) between *P. daejeonensis* and *Pseudocrangonyx* sp. 3 is equivalent to sequence divergence thresholds for discriminating amphipod species (Witt et al. 2006; Rock et al. 2007; Hou et al. 2009). The former is distinguished from the latter in having the following features (features of *Pseudocrangonyx* sp. 3 in parentheses): 1) outerdistal corner of peduncle of pleopod 3 without seta (with seta), 2) each of inner and outer ramus of pleopod 3 2-articulate (3-articulate), 3) outer rami of uropods 1 and 2 without marginal robust setae (with marginal seta), and 4) robust setae on distal part of proximal article of uropod 3 short, not reaching tip of terminal article (long, exceeding tip of terminal article) (Tomikawa pers. observation).

## Supplementary Material

XML Treatment for Pseudocrangonyx daejeonensis
